# Dissecting the molecular mechanism underlying the intimate relationship between cellulose microfibrils and cortical microtubules

**DOI:** 10.3389/fpls.2014.00090

**Published:** 2014-03-13

**Authors:** Lei Lei, Shundai Li, Logan Bashline, Ying Gu

**Affiliations:** Department of Biochemistry and Molecular Biology, Pennsylvania State University, University ParkPA, USA

**Keywords:** cellulose synthase complex, cell wall, microtubules, CESA interactive proteins, cell expansion

## Abstract

A central question in plant cell development is how the cell wall determines directional cell expansion and therefore the final shape of the cell. As the major load-bearing component of the cell wall, cellulose microfibrils are laid down transversely to the axis of elongation, thus forming a spring-like structure that reinforces the cell laterally and while favoring longitudinal expansion in most growing cells. Mounting evidence suggests that cortical microtubules organize the deposition of cellulose microfibrils, but the precise molecular mechanisms linking microtubules to cellulose organization have remained unclear until the recent discovery of cellulose synthase interactive protein 1 , a linker protein between the cortical microtubules and the cellulose biosynthesizing machinery. In this review, we will focus on the intimate relationship between cellulose microfibrils and cortical microtubules, in particular, we will discuss microtubule arrangement and cell wall architecture, the linkage between cellulose synthase complexes and microtubules, and the feedback mechanisms between cell wall and microtubules.

## INTRODUCTION

Microtubules were first observed in plant cells and have been characterized as essential components of the cell division apparatus (Ledbetter and Porter, 1963). Microtubules are present in all eukaryotic cells and are important for cell division, cell expansion, and cell morphogenesis. In contrast to yeast and animal cells, plant cells do not have well-defined microtubule organizing centers such as the centrosomes of animal cells and the spindle pole bodies of yeast cells (Vaughn and Harper, 1998). In post-mitotic plant cells, nucleation of new microtubules occurs at dispersed sites at the cell cortex, the area that is in very close proximity to the plasma membrane within the cell (Nakamura et al., 2010). The microtubules of the plant cortex are arranged into a cortical array, a feature that is unique to plants. Cortical microtubules migrate across the cortex by means of a hybrid treadmilling mechanism, which consists of intermittent depolymerization at the lagging end and polymerization-biased dynamic instability at the leading end (Shaw et al., 2003). The unique behavior of cortical microtubules determines the overall organization of the cortical microtubule array and thereby determines the asymmetric cell growth of plant cells.

In addition, plant cell shape is largely dictated by the opposing forces of turgor pressure and cell wall tension (Skotheim and Mahadevan, 2005; Dumais and Forterre, 2012). Cellulose microfibrils of the cell wall are the major load-bearing component that supports the cell wall tension and that enforces asymmetric cell expansion (Green, 1962). Cellulose microfibrils, composed of β-1, 4-linked glucan chains, are laid down outside of the plasma membrane of plant cells (Somerville, 2006; Carpita, 2011; Endler and Persson, 2011). In cylindrical fast growing cells, cellulose microfibrils are mostly arranged in a transverse orientation that is perpendicular to the axis of elongation. As a consequence of transversely oriented cellulose microfibrils, radial cell expansion is restricted while longitudinal cell expansion is promoted (Roelofsen and Houwink, 1951, 1953). Green’s hypothesis of plant cellular morphogenesis states that the shape of plant cells is determined by the orientation of cortical microtubules because the orientation of newly synthesized cellulose microfibrils is dictated by the cortical microtubule array (Green, 1962). In support of Green’s hypothesis, disruption of either microtubules or cellulose microfibril organization by pharmacological or genetic means leads to cell expansion defects (Hepler and Palevitz, 1974; Itoh, 1976; Hardham and Gunning, 1979; Arioli et al., 1998; Fagard et al., 2000; Lane et al., 2001; Cano-Delgado et al., 2003). Despite the observation that cellulose microfibrils co-align with cortical microtubules, mechanistic details regarding how microtubules and cellulose microfibrils work together to effect cell expansion are lacking. Together with genetic and biochemical methods, recent developments in the live imaging of fluorescent protein-tagged cellulose synthase (CESA) proteins and tubulin isoforms has provided unprecedented opportunities to dissect the molecular mechanisms underlying the intimate relationship between cellulose microfibrils and cortical microtubules.

## THE MACHINERY FOR CELLULOSE BIOSYNTHESIS

In higher plants, cellulose microfibrils are synthesized at the plasma membrane by transmembrane protein complexes, known as cellulose synthase complexes (CSCs; Somerville, 2006). CSCs were initially visualized by freeze fracture electron microscopy in vascular plants as hexagonal rosettes (Mueller and Brown, 1980; Haigler and Brown, 1986). Immunogold labeling studies have shown that these rosettes contain CESA proteins, the only verified component of CSCs in higher plants (Herth, 1983; Kimura et al., 1999). Although the exact composition and stoichiometry of CSCs remains to be discerned, the most popular model predicts that each of the six rosette subunits contains six individual CESA proteins. Assuming that each CESA protein is enzymatically active and synthesizes a single glucan chain, this model would suggest that each CSC could synthesize an elementary cellulose microfibril comprised of 36 glucan chains. However, using advanced techniques in spectroscopy and microscopy, recent measurements of elementary cellulose microfibrils in both primary and secondary cell walls indicate that an 18 or 24-glucan chain model best fits the size of an elementary fiber (Fernandes et al., 2011; Thomas et al., 2013; Zhang et al., 2013). These measurements suggest that either there are less than 36 CESA proteins in each CSC or that not all CESAs of a single CSC are enzymatically active.

In *Arabidopsis*, there are 10 *CESA *genes (*CESA1–10*; Holland et al., 2000; Richmond, 2000). Analyses of mutants with xylem cell defects have revealed that CESA4, CESA7, and CESA8 are each required for cellulose biosynthesis of secondary cell walls (Taylor et al., 2000, 2003). A similar requirement for three different CESA proteins exists in cellulose biosynthesis in primary cell walls (Arioli et al., 1998; Scheible et al., 2001; Beeckman et al., 2002; Burn et al., 2002; Desprez et al., 2002). For cellulose synthesis of primary cell walls, CSCs are composed of CESA1, CESA3, and CESA6 or CESA6-like proteins (CESA2, CESA5, and CESA9; Desprez et al., 2007; Persson et al., 2007). The distinction between primary and secondary CESAs might not be as strict as initially defined (Lei et al., 2012b). For example, CESA7 can partially rescue the growth defect of *cesa3^je5^* when under the expression of the CESA3 promoter (Carroll et al., 2012). Similarly, CESA1can partially rescue the phenotype of the *cesa8^irx1^* null mutant when driven by the CESA7 promoter (Carroll et al., 2012; Li et al., 2013). These results suggest that primary CESAs may have structural properties that allow its incorporation into secondary CSCs and vice versa.

## VISUALIZATION OF CELLULOSE SYNTHASE COMPLEXES

Green fluorescent protein (GFP) fused CESA7, the first fluorescent protein tagged CESA, was shown to complement *irx3-1*, a mutant of *CESA7 *(Gardiner et al., 2003). CESA7-GFP formed thick bands that marked the sites of cell wall deposition in the developing xylem of *Arabidopsis *(Gardiner et al., 2003). However, several characteristics of developing xylem cells prevented the accurate measurement of CESA dynamics. First, developing xylem cells are embedded deep within seedlings at a focal plane that is near the maximum working distance of confocal lenses and therefore difficult to image clearly. As another obstacle to imaging, the thick banding pattern of CESAs in developing xylem cells prevented the accurate measurement of individual CSC particles. To circumvent these difficulties, a similar strategy was developed to visualize and measure the dynamics of fluorescent protein tagged primary CESAs in epidermal cells that synthesize primary cell walls. In etiolated *Arabidopsis* hypocotyls, functional yellow fluorescent protein (YFP) tagged CESA6 (YFP-CESA6) was visualized as distinct particles at the plasma membrane (Paredez et al., 2006). Fluorescent protein fusions of several additional primary CESAs (CESA1, 3, and 5) have since been developed and visualized using similar approaches (Paredez et al., 2006; Desprez et al., 2007; Bischoff et al., 2011; Miart et al., 2014). FP-tagged CESAs, that presumably represent rosette CSCs, exhibited linear motility in the plane of the plasma membrane, traveling an average speed of 300–350 nm/min. The trajectories of plasma membrane localized FP-CESA particles are predicted to represent the position of newly deposited cellulose microfibrils (Li et al., 2014).

Cellulose synthase complexe rosettes are believed to be assembled in Golgi apparatus due to evidence from electron micrographs that showed rosette structures at the trans face of the Golgi apparatus (Haigler and Brown, 1986) and in vesicles close to the plasma membrane (Giddings et al., 1980). Consistent with these early observations, live cell imaging has shown that both primary and secondary CSCs accumulate in Golgi bodies and in vesicles that are close to the plasma membrane (Gardiner et al., 2003; Paredez et al., 2006; Crowell et al., 2009; Gutierrez et al., 2009). Pausing events of CSC-containing Golgi bodies at cortical microtubules were reported in both etiolated hypocotyls (Crowell et al., 2009) and developing xylems (Gardiner et al., 2003) and were proposed to be associated with the secretion of CSCs to the plasma membrane. However, CSC delivery events that occur independently of Golgi pausing events have also been observed in hypocotyls (Sampathkumar et al., 2013). Recent evidence from the spatiotemporal analysis of primary CESAs during cell plate formation revealed that multiple routes of CSC delivery to the cell plate exist from phragmoplast-associated compartments, from Golgi-derived vesicles, and from direct transfer from the plasma membrane (Miart et al., 2014).

In addition to Golgi and plasma membrane localization, CESA is often associated with intracellular small CESA-containing compartments (SmaCCs) upon induction by osmotic stress or cellulose synthesis inhibitor treatment (Gutierrez et al., 2009). A similar population of CESA-labeled compartments was simultaneously described by another research team and referred to as microtubule-associated cellulose synthase compartments (MASCs), and has since been considered to be a subset of the SmaCC population (Crowell et al., 2009, 2010). SmaCCs/MASCs exhibit extended periods of pausing at cortical microtubules with intermittent instances of rapid motility that is driven by microtubule depolymerization (Crowell et al., 2009; Gutierrez et al., 2009). It has been hypothesized that microtubule-tethered SmaCCs/MASCs may function in the delivery or the internalization of CSCs. After relief from osmotic stress, some CSC delivery events coincided with microtubule-tethered SmaCCs that showed microtubule tip-tracking behavior before the CSC delivery, suggesting that microtubules might control CSC trafficking and delivery to the plasma membrane, although the delivery rate of CSCs to the plasma membrane was unaffected by pharmacological microtubule depolymerization (Gutierrez et al., 2009). Actin also plays a role in controlling the distribution of CSCs during the synthesis of both primary and secondary cell walls. In the epidermal cells of etiolated hypocotyls, treatment with actin depolymerizing agents, Cytochalasin D or Latrunculin B, caused CESA-containing Golgi bodies to aggregate and led to reduction of CSCs at the plasma membrane in areas that were devoid of aggregated Golgi bodies (Crowell et al., 2009; Gutierrez et al., 2009). The distribution of CSCs during secondary cell wall synthesis is also dependent on the actin cytoskeleton. Latrunculin B treatment resulted in a loss of actin filaments that are typically positioned close to CSC bands in xylem cells and consequently resulted in a loss of CSC bands (Wightman and Turner, 2008). These results suggest that the plant cytoskeleton is involved in CSC distribution and trafficking during both primary and secondary cell wall synthesis.

The characterization of primary CSC behavior has been more successful than secondary CSC characterization because of the ease of imaging primary cell wall synthesizing tissues, such as epidermal cells, which are exposed at the surface of the plant, as opposed to secondary cell wall producing tissues, which are typically buried deep within the plant. Cellulose microfibrils in the secondary wall are presumably longer and more bundled than those in the primary cell wall. The production of more bundled microfibrils may be due to an increased clustering of CSCs at distinct sites of the plasma membrane underneath the secondary cell wall. As proof of concept, in algae, it has been proposed that CSC clustering is responsible for the formation of cellulose microfibrils with a diameter of 50 nm, which is indicative of a high degree of microfibril bundling (Hogetsu, 1983; Giddings and Staehelin, 1988). If imaging of secondary cell wall producing cells could be improved, some parameters of secondary CSCs may provide helpful insight into secondary CSC velocity and clustering as well as how these parameters affect the properties of the cellulose microfibril.

## CELLULOSE MICROFIBRIL ORIENTATION AND MICROTUBULE ARRANGEMENT

The presence of cortical microtubules that are adjacent to the plasma membrane is a unique feature of plant interphase cells (Ledbetter and Porter, 1963; Baskin, 2001; Shaw et al., 2003). The formation of organized cortical microtubule arrays is believed to be generated by a self-organizing process that is mainly driven by two characteristics: the treadmilling behavior of microtubules and interactions between microtubules (Dixit and Cyr, 2004a,b). In rapidly elongating cells, such as epidermal cells of the root elongation zone, cortical microtubules uniformly organize into arrays that are perpendicular to the elongation axis of the cell (Sugimoto et al., 2000; Granger and Cyr, 2001). Newly deposited cellulose microfibrils of the cell wall are organized in a similar transverse pattern that mirrors the cortical microtubule array on the inner face of the plasma membrane. The co-alignment between cellulose microfibrils and cortical microtubules suggests that these two molecular components are intimately associated with one another. Interestingly, long before the observation of cellulose microfibril and microtubule co-alignment, the observation that colchicine, a microtubule-depolymerizing reagent, disrupted the organization of newly deposited cellulose microfibrils led to Green’s hypothesis that a cytoplasmic structure (microtubules had not yet discovered) determines the orientation of cellulose microfibrils (Green, 1962; Ledbetter and Porter, 1963; Heath, 1974; Herth, 1980). Since then, cellulose microfibril and microtubule co-alignment has been observed in many types of plant cells, but exceptions have also been documented (Baskin, 2001). The simultaneous live imaging of YFP-CESA-labeled CSCs and CFP-tubulin-labeled microtubules has revealed an intimate association between CSCs and microtubules in which motility of active CSCs follows trajectories that co-align with underlying cortical microtubules in both primary and secondary cell wall synthesizing plant cells (Gardiner et al., 2003; Paredez et al., 2006). In support of the alignment hypothesis, changes in microtubule orientation resulted in a correlated shift in CSC trajectories. Complete removal of microtubules by the microtubule-depolymerizing agent, oryzalin, resulted in a uniform distribution of CSCs (Gardiner et al., 2003; Crowell et al., 2009; Li et al., 2011). Most of the early observations of the co-alignment were made on fixed tissues so that the dynamic features of cortical microtubules were unattainable. With newly developed live cell imaging tools, we can now examine the molecular details and dynamics of the relationship between cellulose and microtubules.

During cell growth, cortical microtubule arrays constantly undergo reorganization due to the dynamic instability of microtubules. A striking example of microtubule reorganization during cell expansion is the rotary movement of the cortical microtubule arrays at the outer surface of epidermal cells of the hypocotyl (Chan et al., 2007). CSC trajectories rotate simultaneously with cortical microtubules. This rotational readjustment of CSC trajectories causes successive layers of cellulose microfibrils to be deposited at progressively varying angles. Pharmacological disruption of the rotary movement of microtubules inhibited the rotation of CESA trajectories, suggesting that microtubules predominantly guide the rotation of CSC trajectories, thereby affecting the multi-angle cellulose deposition during cell wall assembly (Chan et al., 2007, 2010; Chan, 2012). Recently, multiple angles of cellulose microfibrils were observed at the inner, youngest layers of hydrated onion epidermal cell walls using atomic force microscopy (AFM; Zhang et al., 2013). The multi-angle pattern of cellulose microfibrils in successive cell wall layers may be a common feature during anisotropic cell expansion in many cell types. However, in epidermal cells of the root elongation zone in *Arabidopsis*, neither microtubule arrays nor CSC trajectories undergo rotary movement. Instead, in this cell type, the establishment of a multi-angled pattern of cellulose microfibrils in cell wall layers has been proposed to result from passive reorientation of cellulose microfibrils as cell expansion occurs (Lloyd, 2011). In support of this idea, cellulose microfibrils of the root elongation zone that were labeled with Pontamine fast scarlet 4B (S4B) dye were shown to exhibit varying angles, gradually changing the orientation from perpendicular at inner layers to parallel to the elongation axis at outer layers (Anderson et al., 2010). While the biological significance of varying cellulose microfibril orientation in successive cell wall layers is currently unknown, one possible function might be to provide strength and rigidity to the cell wall.

## THE LINKAGE BETWEEN CELLULOSE SYNTHASE COMPLEXES AND MICROTUBULES

Two models have been put forward to explain the alignment between cellulose microfibrils and cortical microtubules: the direct guidance model and the bumper model. The direct guidance model postulates that some type of direct linkage exists between CSCs that are actively synthesizing cellulose and cortical microtubules (Heath, 1974; Somerville, 2006) while the bumper model suggests that cortical microtubules define channels within which active CSCs move at the plasma membrane without any physical link between the CSCs and the cortical microtubules (Giddings and Staehelin, 1991). One important quality of a linker protein between CSCs and microtubules is the ability to interact with microtubules. In *Arabidopsis*, many microtubule-associated proteins and microtubule motor proteins have been identified. One such protein, the fragile fiber 1 (FRA1) kinesin motor protein, was proposed to be a possible linker protein between CSCs and microtubules due to the abrogation of cellulose microfibril organization in secondary cell walls of fiber cells in the *fra1 *mutant (Zhong et al., 2002). However, further characterization of FRA1 suggests that FRA1 does not act as a CSC-microtubule linker protein. In an *in vitro* analysis, the motor domain of FRA1 was observed to travel along microtubules at a velocity that is about 100 times faster than the average velocity of CSC movement (Zhu and Dixit, 2011). Moreover, FRA1 exhibited unidirectional movement toward the plus end of microtubules while CSCs move bidirectionally along microtubules.

Aside from being able to interact with microtubules, a CSC-microtubule linker protein must also have the ability to interact (directly or indirectly) with the CSC. In an attempt to identify candidates that interact with CESAs, a yeast two-hybrid screen was performed using the central cytosolic domain of primary CESAs (Gu and Somerville, 2010; Gu et al., 2010). Cellulose synthase interactive protein 1 (CSI1) was identified among several dozen putative CESA-interacting proteins. Consistent with CSI1 playing a role in cellulose biosynthesis, *csi1* null mutants displayed a reduction in crystalline cellulose content and reduced anisotropic cell expansion in *Arabidopsis* hypocotyls and roots (Gu et al., 2010). Several lines of evidence suggest that CSI1 is a linker between active CSCs and cortical microtubules. CSI1 interacted with CESA3 and CESA6 in a split-ubiquitin yeast two-hybrid assay and CSI1 interacted with microtubules in an *in vitro* microtubule-binding assay (Li et al., 2011; Lei et al., 2012a). *In planta*, fluorescent protein-tagged CSI1 co-localized with CESA3 and CESA6 and traveled together with CESA3 and CESA6 along trajectories that co-aligned with cortical microtubules and at velocities that are typical of active CSCs (Gu et al., 2010; Li et al., 2011; Lei et al., 2012a). Furthermore, the association between CSCs and microtubules was disrupted in *csi1 *mutants, suggesting that CSI1 is essential for the alignment between CSC trajectories and cortical microtubules.

In addition to its essential role in associating CSCs with microtubules, CSI1 is also critical in maintaining the normal dynamics of CSCs. CSCs move along cortical microtubules at an average velocity of 300–350 nm/min in the epidermal cells of etiolated *Arabidopsis *hypocotyls (Paredez et al., 2006; Gu et al., 2010; Li et al., 2011). In *csi1* null mutants, the average CSC velocity was reduced to about 70% of that of wild type (Gu et al., 2010; Li et al., 2011). Although a 200 nM dose of oryzalin, a microtubule-depolymerizing drug, had no affect on CSC velocity in hypocotyls (Chan et al., 2010), a prolonged 20 μM dose of oryzalin reduced the velocity of CSCs to a similar extent as that in *csi1* mutants. However, the removal of microtubules also affects the localization of CSI1 so it is not clear whether the CSC velocity reduction is influenced solely by the loss of microtubules or due to compromised CSI1 function (Li et al., 2011). Recent studies suggest that CSC velocity is correlated with cellulose crystallinity. For example, a point mutation in the catalytic region of CESA1 (*cesa1^D604N^*) reduces CSC velocity and crystallinity (Fujita et al., 2013). In *mor1* mutants where the total microtubule mass is reduced, cellulose crystallinity and CSC velocity remain high (Fujita et al., 2011). While the mechanism of the influence of CSI1 on CSC velocity remains unknown, evidence suggests that microtubules are capable of regulating CSC velocity. In etiolated *cesa6^prc1^^-^^1^* hypocotyls, the removal of cortical microtubules led to a significant increase in GFP-CESA5 velocity (Bischoff et al., 2011). In another case, the asymmetric distribution of CSC velocity directionality caused by the expression of a CESA1 variant was shown to be dependent on the presence of cortical microtubules (Chen et al., 2010). Presumably, the removal of microtubules also disrupts the function of microtubule-associated proteins. Therefore, it is likely that microtubules together with microtubule-associated components contribute to regulating the velocity of CSCs. The molecular mechanism by which CSI1 remains associated to both CSCs and microtubules is also of special interest because both of these components are highly dynamic. There are two CSI1-like proteins in *Arabidopsis*, referred to as CSI2 and CSI3. CSI1 shares about 60% sequence similarity with CSI2 and CSI3. Promoter::GUS transcriptional analyses revealed that CSI3 was expressed in many tissues while CSI2 expression was undetectable (Lei et al., 2013). Similar to CSI1, CSI3 interacted with multiple primary CESAs in a split-ubiquitin yeast two-hybrid assay and CSI3 co-localized with CSCs and traveled along cortical microtubule tracks at comparable velocities. However, *csi3* null mutants did not display any defect in cell expansion nor did *csi3* affect the CSC velocity or the co-alignment of CSCs and microtubules. The functional difference between CSI1 and CSI3 was further supported by the inability of *ProCSI1:GFP-CSI3* to complement the phenotype of* csi1-3*. Although *csi3* mutants lack an apparent phenotype, *csi1csi3 *double mutants displayed enhanced cellulose biosynthesis-related phenotypes, suggesting that CSI3 plays a role in cellulose biosynthesis (Lei et al., 2013).

While CSI1 was shown to mediate the interaction between active CSCs and cortical microtubules at the plasma membrane, CSI1 was also shown to label cortically localized SmaCCs/MASCs, indicating that CSI1 is potentially involved in CESA trafficking and/or delivery to the plasma membrane (Bringmann et al., 2012; Lei et al., 2012a). Interestingly, CSI1 puncta only localize to the plasma membrane and cortical region so CSI1 does not localize to CESA-containing Golgi bodies. Therefore, it is likely that CSI1 only associates with CSCs after they are fully assembled and within proximity to the plasma membrane. It remains to be determined how CSI1 is recruited to the plasma membrane and how CSI1 mediates the association between CSCs and cortical microtubules. The recruitment of the CSI1 protein to the plasma membrane may be the function of the C-terminal C2 domain of CSI1. The first identification of a C2 domain occurred using a membrane-associated protein kinase C, and many C2 domains have been shown to target proteins to cell membranes by binding to phospholipids in a calcium-dependent or independent manner (Davletov and Sudhof, 1993; Ochoa et al., 2001; Rickman and Davletov, 2003). Consistent with the role of the CSI1 C2 domain in targeting the CSI1 protein to the plasma membrane, a C2 domain deletion variant of CSI1, YFP-CSI1ΔC2, did not complement the *csi1 *mutant phenotype, nor did it localize to CESA complexes at the plasma membrane (Bringmann et al., 2012).

**FIGURE 1:**
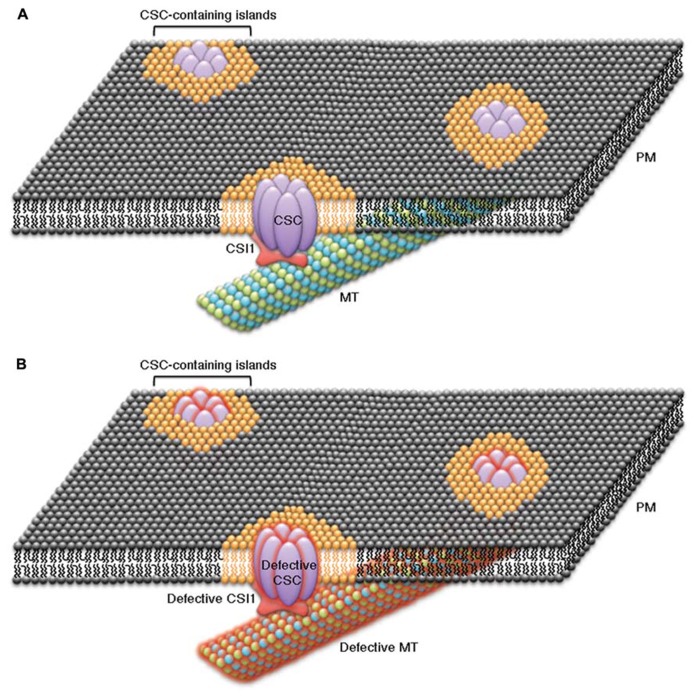
**Schematic representation of the cellulose biosynthesis machinery displays a continuum between cellulose synthase complexes (CSCs), CSC-associated proteins (e.g., CSI1), cortical microtubules (MT), and the plasma membrane.** The transmembrane CSCs might be embedded in lipid islands or lipid micro-domains of specialized lipid content (orange lipids) that are surrounded by a sea of plasma membrane of normal composition (black lipids). CSI1 might act as a scaffold between CSCs, microtubules, and specialized lipids of CSC-containing islands by interacting with all three of these components to form a CSC, CSI1, microtubule, and lipid continuum. **(A)** The components in the continuum, including CSCs, CSC-interacting proteins, specialized lipids, and cortical microtubules, are closely associated. Under normal conditions CSCs that are within CSC-containing islands move along cortical microtubules during cellulose biosynthesis in a CSI1-dependent manner. **(B)** A defect in any single component of the CSC continuum causes a disruption of each of the other components of the continuum. Examples of experimental evidence that supports the CSC continuum model include: disruption of cortical microtubules influences the distribution and dynamics of CSCs and CSI1, genetic disruption of CSI1causes changes in both CSCs and cortical microtubules, and genetic disruption of CSCs or CSC-associated proteins (e.g., KOR1) affect the organization of cortical microtubules.

The putative lipid-binding activity of the C-terminal C2 domain of CSI1 may also allow CSI1 to influence the organization lipid micro-domains that contain CSCs at the plasma membrane. Studies in mammalian cells, have shown that lipids and proteins are not uniformly distributed at the plasma membrane, but instead specialized lipid environments can be organized into discrete islands or micro-domains and certain proteins prefer to be partitioned into these specialized lipid environments (Simons and van Meer, 1988; van Meer and Simons, 1988). CSCs are large transmembrane complexes and have been speculated to form membrane micro-domains together with specific lipids and other associated proteins (Guerriero et al., 2010; **Figure 1A**). If CSCs are partitioned into islands of special lipid content, some properties of CSC-containing islands, such as membrane fluidity, may differ from the properties of the surrounding plasma membrane. Cortical microtubules have been proposed to direct the formation of plasma membrane micro-domains that could influence the activities of CSCs (Fujita et al., 2012; Schrick et al., 2012). It is possible that a relationship exists between CSI1, CSCs, cortical microtubules, and specialized lipid micro-domains to provide a mechanism for microtubule-dependent organization of CSC-containing islands in which the proper function of CSCs is contingent on the integrity of each of these components (**Figure 1B**). Several lines of evidence are consistent with this model. First, disruption of the cortical microtubules affected the distribution and dynamics of both CSCs and CSI1 puncta (Li et al., 2011). Second, lack of CSI1 in *csi1 *null mutants led to defects in both CSCs and cortical microtubules (Li et al., 2011; Bringmann et al., 2012; Mei et al., 2012; Landrein et al., 2013). Third, mutants with defective CSCs or CSC associated proteins affected the organization of cortical microtubules (Paredez et al., 2008). Further evidence must be obtained to validate the existence of specialized CSC-containing lipid islands and the dependency of these structures on the integrity of CSCs, CSI1, and cortical microtubules.

## FEEDBACK MECHANISM BETWEEN CELL WALL AND CYTOSKELETON

The concept of a “dynamic reciprocity” between the intracellular cytoskeleton and the extracellular matrix (ECM) was first postulated in reference to mammalian cells (Edelman, 1983). Despite the different composition of the mammalian ECM and the plant cell wall, it has been postulated that plant cells might regulate the perception and transduction of positional information using similar sensing mechanisms that involve a feedback interaction between the cell wall and the cytoskeleton (Wyatt and Carpita, 1993). Although plants lack the counterparts of most of the mammalian components involved in the relationship between the cytoskeleton and the ECM, several lines of evidence suggest that feedback exists between the cell wall and the cytoskeleton. For example, physically separating the cell wall from the plasma membrane by plasmolysis induced microtubule disintegration, suggesting that a physical connection between the plasma membrane and the cell wall is important for microtubule organization (Komis et al., 2002). Both pharmacological and genetic studies have shown that feedback from the cell wall regulates microtubule organization. Isoxaben, a cellulose biosynthesis inhibitor, caused reorientation of microtubules in plant cells (Fisher and Cyr, 1998; Himmelspach et al., 2003; Paredez et al., 2008). The reorganization of cortical microtubules upon isoxaben treatment can be attributed to a reduction in CSC activity since isoxaben treatment depleted CSCs from the plasma membrane (Gutierrez et al., 2009). Two cellulose biosynthesis deficient mutants, a null allele of *CESA6* and a new allele of *KORRIGAN (KOR)*, were identified in a screen for *Arabidopsis* mutants that are hypersensitive to oryzalin, a microtubule-depolymerizing drug. Both *kor1-3* and *cesa6^prc1^^-^^20^* exhibited altered orientation and stability of cortical microtubules in root cells and reduced CSC velocity (Paredez et al., 2008). Together, the observations that CSC velocity is reduced in cases where either CESA6, KOR, or microtubules are missing and that *kor1–3* and *cesa6^prc1^^-^^20^* mutants affect microtubule organization, supports the idea that a two-way feedback regulation mechanism exists between the cytoskeleton and the cell wall. Since attempts to purify integrin-like and spectrin-like proteins in plants using heterologous probes and searches for genes with sequence homology have been unsuccessful, the components involved in feedback between the cell wall and the cytoskeleton in plants may be unconventional (Nick, 2013). The function of cortical microtubules in plant cells is certainly not limited to regulating cellulose synthesis, so the feedback between microtubules and the cell wall may potentially be integrated with other microtubule-related functions. The unique dynamic features of microtubules add another layer of complexity to the investigation of the feedback regulation between the cytoskeleton and the cell wall in plants.

## Conflict of Interest Statement

The authors declare that the research was conducted in the absence of any commercial or financial relationships that could be construed as a potential conflict of interest.
